# Differential protein expression in the hippocampi of resilient individuals identified by digital spatial profiling

**DOI:** 10.1186/s40478-022-01324-9

**Published:** 2022-02-14

**Authors:** Jamie M. Walker, Shiva Kazempour Dehkordi, Anna Fracassi, Alison Vanschoiack, Anna Pavenko, Giulio Taglialatela, Randall Woltjer, Timothy E. Richardson, Habil Zare, Miranda E. Orr

**Affiliations:** 1 Department of Pathology and Laboratory Medicine, University of Texas Health Science Center, San Antonio, TX USA; 2 Department of Pathology, Glenn Biggs Institute for Alzheimer’s & Neurodegenerative Diseases, University of Texas Health Science Center, 7703 Floyd Curl Dr., MC 8070, San Antonio, TX 78229-3900 USA; 3 Department of Cell Systems and Anatomy, University of Texas Health San Antonio, San Antonio, TX USA; 4 Mitchell Center for Neurodegenerative Diseases, Department of Neurology, UTMB, Galveston, TX USA; 5 NanoString Technologies, Seattle, WA USA; 6 Department of Pathology & Laboratory Medicine, Oregon Health and Science University, Portland, OR USA; 7 Section of Gerontology and Geriatric Medicine, Department of Internal Medicine, Wake Forest School of Medicine, Winston-Salem, NC 27157 USA; 8 Sticht Center for Healthy Aging and Alzheimer’s Prevention, Wake Forest School of Medicine, Winston-Salem, NC 27157 USA; 9 Salisbury VA Medical Center, Salisbury, NC 28144 USA; 10 Department of Internal Medicine, Wake Forest School of Medicine, 575 Patterson Ave, Winston-Salem, NC 27101 USA

**Keywords:** Alzheimer disease, Neurofibrillary tangles, Resilient, Digital spatial profiling (DSP), Hippocampus, Senescence

## Abstract

Clinical symptoms correlate with underlying neurodegenerative changes in the vast majority of people. However, an intriguing group of individuals demonstrate neuropathologic changes consistent with Alzheimer disease (AD) yet remain cognitively normal (termed “resilient”). Previous studies have reported less overall neuronal loss, less gliosis, and fewer comorbidities in these individuals. Herein, NanoString GeoMx™ Digital Spatial Profiler (DSP) technology was utilized to investigate protein expression differences comparing individuals with dementia and AD neuropathologic change to resilient individuals. DSP allows for spatial analysis of protein expression in multiple regions of interest (ROIs) on formalin-fixed paraffin-embedded sections. ROIs in this analysis were hippocampal neurofibrillary tangle (NFT)-bearing neurons, non-NFT-bearing neurons, and their immediate neuronal microenvironments. Analyses of 86 proteins associated with CNS cell-typing or known neurodegenerative changes in 168 ROIs from 14 individuals identified 11 proteins displaying differential expression in NFT-bearing neurons of the resilient when compared to the demented (including APP, IDH1, CD68, GFAP, SYP and Histone H3). In addition, IDH1, CD68, and SYP were differentially expressed in the environment of NFT-bearing neurons when comparing resilient to demented. IDH1 (which is upregulated under energetic and oxidative stress) and PINK1 (which is upregulated in response to mitochondrial dysfunction and oxidative stress) both displayed lower expression in the environment of NFT-bearing neurons in the resilient. Therefore, the resilient display less evidence of energetic and oxidative stress. Synaptophysin (SYP) was increased in the resilient, which likely indicates better maintenance of synapses and synaptic connections. Furthermore, neurofilament light chain (NEFL) and ubiquitin c-terminal hydrolase (Park5) were higher in the resilient in the environment of NFTs. These differences all suggest healthier intact axons, dendrites and synapses in the resilient. In conclusion, resilient individuals display protein expression patterns suggestive of an environment containing less energetic and oxidative stress, which in turn results in maintenance of neurons and their synaptic connections.

**Supplementary Information:**

The online version contains supplementary material available at 10.1186/s40478-022-01324-9.

## Introduction

Alzheimer disease neuropathologic change (ADNC) levels of intermediate and high are considered to be sufficient to explain cognitive impairment by current consensus criteria [36]. However, it has been reported that up to 70% of cognitively normal (CN) individuals have some degree of AD pathology at death and about 30% of these CN individuals meet the criteria for intermediate or high ADNC [1, 7, 8, 23, 58]. These CN individuals with intermediate to high ADNC are often described as “cognitively resilient.” Investigations into the unique characteristics of these resilient people have ranged from studies of their clinical and lifestyle differences [1, 10, 21, 42], neuropathologic differences [26, 43, 50], genetic differences [15, 47], brain imaging and metabolic signatures [2, 41, 51], as well as their distinct synaptic characteristics and cytokine profiles [6, 44, 63]. Findings from these studies have revealed higher education levels in the resilient, more engagement in physical and social/mental activities [1, 10, 42], fewer neuropathologic comorbidities [1, 26, 43, 50], single nucleotide polymorphisms (SNPs) related to immune response, as well as vascular, metabolic and mental health being associated with resilience against amyloidosis [15, 47], imaging and metabolic signatures suggesting better maintenance of structure (cortical thickness) and function (FDG-PET) despite AD pathologic change, especially in the anterior cingulate and temporal pole [2, 41, 51], and synapses with fewer tau and Aβ-oligomers in the post-synaptic densities and less ApoE in the pre-synaptic synaptosomes in the resilient [44, 54, 63].

In the neurodegenerative field, one of the only studies published to date which utilized NanoString GeoMx™ DSP, analyzed differential protein expression in and around plaques in cases with AD pathology compared to AD cases with *TREM2* variations, using amyloid plaques and their immediate microenvironments as ROIs [46]. They found differences in the AD and TREM2 cases which included increased p-tau in the microenvironment of the plaques of the TREM2 variant carriers, as well as increased NMDAR1 and S100B in the center of the plaques. Given that NFT burden correlates more closely with cognitive status [53], we used a similar approach, but focused on neurons with and without NFTs.

Herein, we utilized the GeoMx DSP technology to compare NFT-bearing neurons to non-NFT-bearing neurons (as well as their immediate neuronal microenvironments) in cases with AD pathology. Furthermore, we compare NFT-bearing neurons in resilient individuals (non-demented with AD pathology) to NFT-bearing neurons in individuals with dementia and AD pathology, and their immediate neuronal microenvironments, as well as the non-NFT-bearing neurons in these same subgroups.

## Materials and methods

### Cases

We obtained posterior hippocampal sections at the level of the lateral geniculate nucleus (LGN) from 14 patients with histopathologic findings of Alzheimer disease (Table 1). These included 6 demented and 8 resilient, defined using the standard Mini-Mental State Examination (MMSE) and provided cognitive status from The Mitchell Center for Neurodegenerative Diseases at University of Texas Medical Branch and Department of Pathology at Oregon Health and Science University using standard protocols. For our purposes, “demented” was defined as MMSE < 19, and “resilience” was defined as MMSE ≥ 19 with Braak neurofibrillary tangle stage ≥ IV, and age ≥ 87-years-old.

**Table 1:** Case demographics, cognitive status, and AD neuropathologic changes

Case Number	Subgroup	Cognitive Status	MMSE	Age at Death (years)	Sex	PMI (hours)	AD Neuropathologic Change		
							Braak	Thal	CERAD
1	DEM	Demented	11	86	M	16	VI	5	2
2	DEM	Demented	< 19	85	F	5	VI	5	2
3	DEM	Demented	< 19	63	F	44	VI	5	3
4	DEM	Demented	< 19	67	F	13	VI	5	3
5	DEM	Demented	< 19	71	F	17	VI	5	2
6	DEM	Demented	< 19	74	F	2	VI	4	3
7	RES	MCI	23	87	F	6	IV	4	1
8	RES	MCI	19	103	M	10	IV	2	1
9	RES	Normal	26	94	F	9	IV	5	2
10	RES	Normal	29	93	F	15.5	IV	4	2
11	RES	Normal	27	> 89	F	48	IV	4	1
12	RES	Normal	29	87	F	3	IV	4	1
13	RES	Normal	27	> 89	F	4.5	VI	4	3
14	RES	Normal	26	> 89	F	8	VI	4	1

## NanoString GeoMx™ DSP

**Fig. 1:**
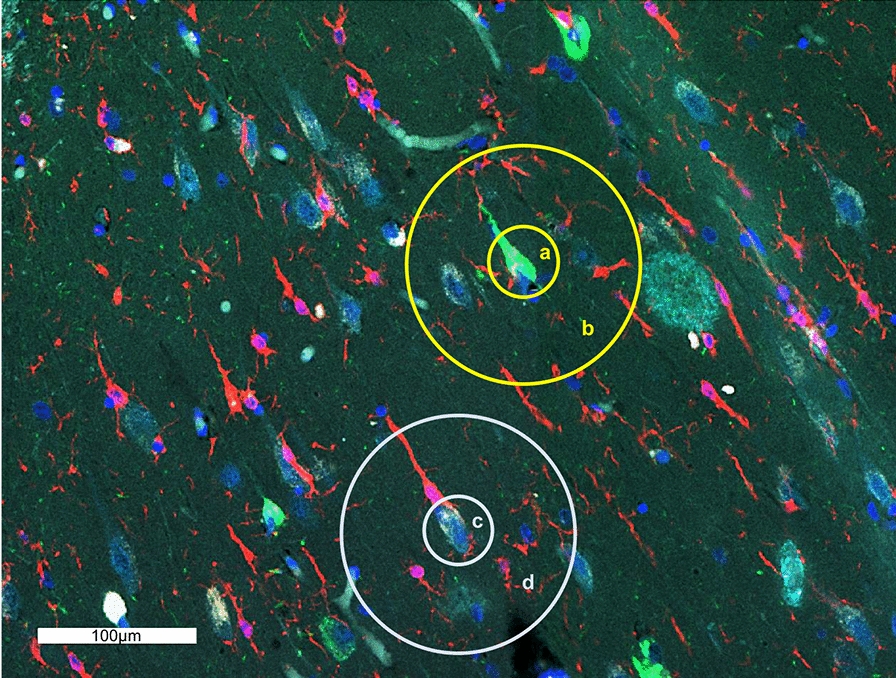
Hippocampal CA1 subregion labeled with the four morphology markers (AT8, green; β-amyloid, aqua; IBA-1, red; nuclear marker SYTO13, blue) and representative regions of interest (ROIs), including **a** NFT-bearing neuron and **b** the immediate neuronal microenvironment of and NFT, **c** normal non-tangle-bearing neuron, and **d** immediate neuronal microenvironment of a normal neuron, scale bar represents 100 µm

Formalin-fixed paraffin-embedded (FFPE) hippocampal sections were deparaffinized and incubated with a cocktail containing 86 antibodies (Additional file 1), each of which are conjugated to unique UV-photocleavable oligonucleotide tags. Specific fluorescently-labeled antibodies were used as the morphology markers to select regions of interest (ROIs), according to the manufacturer’s instructions using a kit and protocols developed specifically for DSP protein assays (https://www.nanostring.com/products/geomx-digital-spatial-profiler/geomx-protein-assays/). In this study, NFTs, β-amyloid plaques, and microglia were selected as the morphology markers and identified visually by fluorescently-labeled antibodies against AT8 (phospho-tau—Ser202,Thr205) (ThermoFisher Scientific, MN1020, conjugated with AF594 using Alexa Fluor 594 antibody Labeling Kit from Thermo A20185—green), β-amyloid (MOAB-2) (Alexa Fluor 532—aqua) (Novus Biologicals NBP2-1307AF532) and IBA-1 (20A12.1) (Alexa Fluor 647—red) (Millipore Sigma MABN92-AF647). Nuclei were identified by staining with Syto13 (ThermoFisher Scientific), a nucleic acid-binding blue fluorescent dye. In each case, four neurofibrillary tangles in the CA1 subregion of the hippocampus and two non-tangle-bearing (normal) neurons were selected as ROIs for high-resolution multiplex profiling, as well as their respective surrounding microenvironments comprising a total of 12 ROIs per slide. A 10-μm in diameter circle was selected as the center ROI surrounding each neuron, and a 50-μm in diameter concentric circle around the center ROI was selected for the immediate neuronal microenvironment, excluding the central 10-μm region. Overall, we compared 24 demented NFTs to 32 resilient NFTs in one analysis, and 56 NFTs to 28 normal neurons in another analysis, as well as their immediate microenvironments. Examples of our ROIs are demonstrated in Fig. 1. We avoided neurons and NFTs near Aβ plaques. All ROIs were analyzed using an early version of NanoString’s GeoMx™ Digital Spatial Profiler system (NanoString Technologies, Seattle, WA, USA), as described previously [33]. Briefly, in this process, the selected ROIs were illuminated individually via UV light on the GeoMx™ DSP, photocleaving the oligonucleotides from the antibodies bound in the ROIs. The oligonucleotides were collected on a 96-well microwell plate. Individual microwells contain the collected photocleaved oligonucleotides from each spatially resolved ROI. These oligonucleotides were then hybridized to four-color, six-spot optical barcodes and analyzed on the nCounter® platform, resulting in distinct spatially mapped counts that correspond to the amount of each antibody that was present in each ROI. Digital counts were first normalized with internal controls (GAPDH, Histone H3, and S6) for system variation, although these have been shown to have altered expression in neurofibrillary tangles in Alzheimer disease [25, 27], therefore, samples were normalized to spiked-in External RNA Controls Consortium (ERCC) RNA control counts [45].

## DSP analysis and statistics

The raw data included expression levels of 86 proteins and 3 IgG controls in 12 ROIs from each of the 14 cases. Raw data was normalized to ERCC RNA control counts. Data included 6 demented and 8 non-demented (resilient) cases. The mean and median of age were 84 and 87 years old, respectively, with a standard deviation of 11 years. In order to identify the differentially expressed proteins in each comparison, we used the *limma* package (Version 3.48.1) [49] and custom R (Version 4.1.0) scripts [57]. The linear regression model was adjusted for sex, age and age squared. The correlation between technical replicates was estimated using duplicateCorrelation [55] function and then added to the linear model. The p-values were adjusted for multiple analyses using Benjamini–Hochberg with a false discovery rate (FDR) of 0.01. The heatmaps were generated using ComplexHeatmap (Version 2.8.0) [20].

## Results

### NFT-bearing neurons vs non-NFT-bearing neurons

**Fig. 2:**
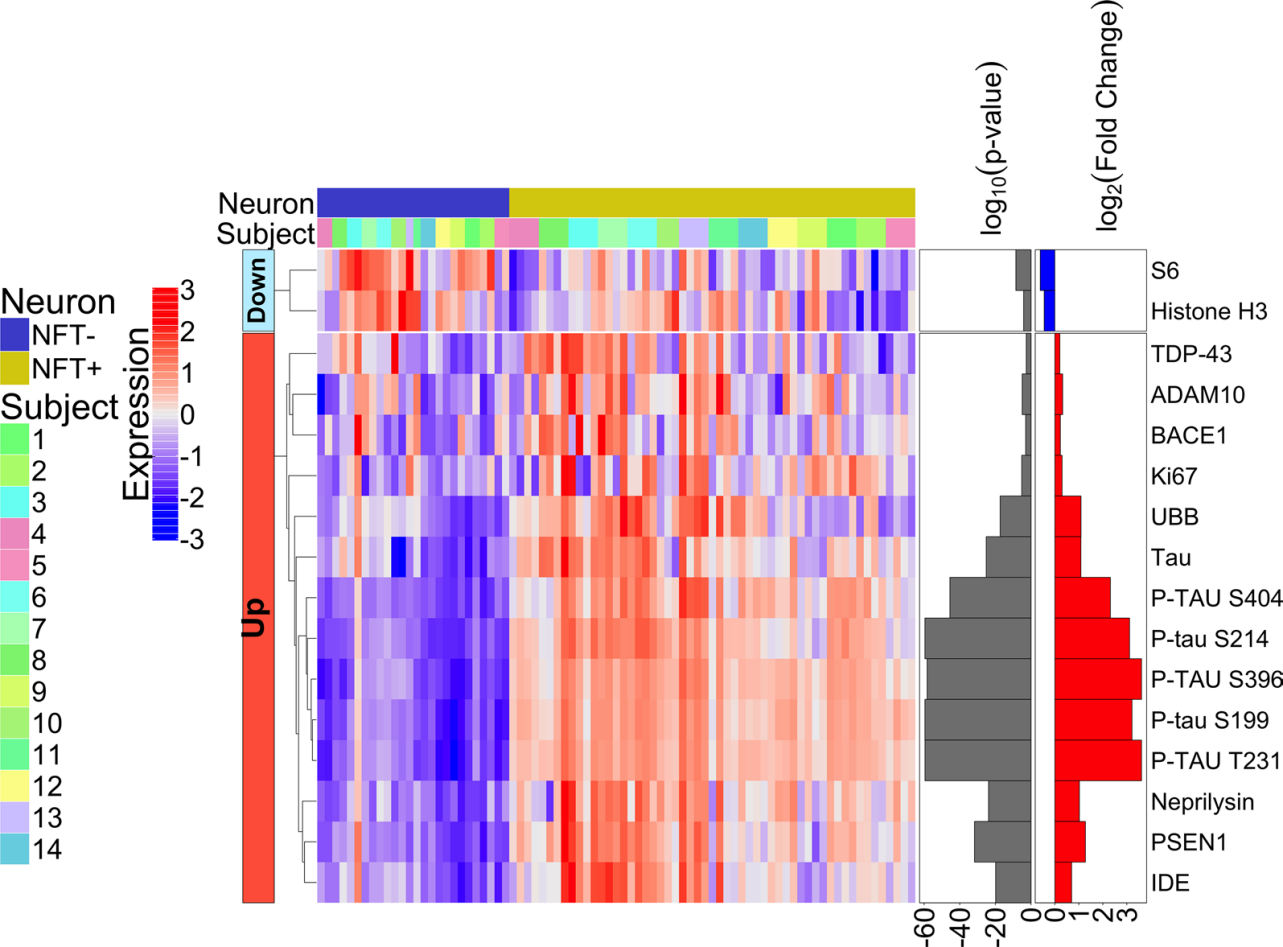
Heatmap of differentially expressed proteins when comparing NFTs to normal non-NFT-bearing neurons (*p* < 0.01); FDR was used to adjust the p-values and it is represented as log10 (*p *value) in the figure with a threshold of 0.01

Our first analysis of the DSP data compared NFT-bearing neurons (n = 56) to non-NFT-bearing neurons (n = 28) in the CA1 subregion of the hippocampus, using all cases (demented and resilient). Out of the 86 proteins analyzed, 16 were differentially expressed between the neuron groups (*i.e.,* with NFTs versus without NFTs) (Fig. 2, Additional file 2). As expected, levels of total and phosphorylated-tau were increased in the NFT-bearing neurons (*i.e.,* Ser404, Ser214, Ser296, Ser199a and Thr231). Other proteins upregulated in NFT-bearing neurons included those known to be involved in β-amyloid processing including PSEN1 (Presenilin 1, a subunit of the γ-secretase complex that cleaves APP); BACE1 (β-secretase 1, a β-secretase responsible for the first step in the proteolytic cleavage of APP); ADAM10 (A Disintegrin and Metalloproteinase, an α-secretase in neurons responsible for cleaving APP into a neuroprotective fragment that is not capable of plaque formation); neprilysin (CD10, a protease that inactivates several peptide hormones and is involved in the rate limiting step of β-amyloid degradation) and IDE (insulin degrading enzyme, a protease capable of degrading monomeric Aβ). Upregulation of PSEN1 and BACE1 may increase APP processing and downstream Aβ production and deposition, whereas upregulation of ADAM10, neprilysin, and IDE may be protective as they would help degrade the Aβ. We note that the elevated expression of APP and its processing machinery resulted in a modest, non-significant, increase in Aβ42 in NFT-bearing neurons (Additional file 2) suggesting a near balance was met by upregulating both Aβ producing (*i.e.,* APP, PSEN1, BACE1) and degrading proteins *(i.e.,* ADAM10, neprilysin and IDE)*.*

Other notable differentially expressed proteins included Histone H3 and Ki-67. Their expression patterns in NFT-bearing neurons (lower and higher, respectively) is consistent with chromatin remodeling and potential exit from quiescence [40] which have been linked to pathogenic tau accumulation [18] and senescence [13, 37]. We also found elevated TDP-43 and ubiquitin in NFT-bearing neurons. These findings may signify a dysfunctional ubiquitin proteasome system, which has been mechanistically linked to tauopathy [38] resulting in the accumulation of multiple proteins (*i.e.,* tau, TDP-43 and UBB) in NFT-bearing neurons.

**Fig. 3:**
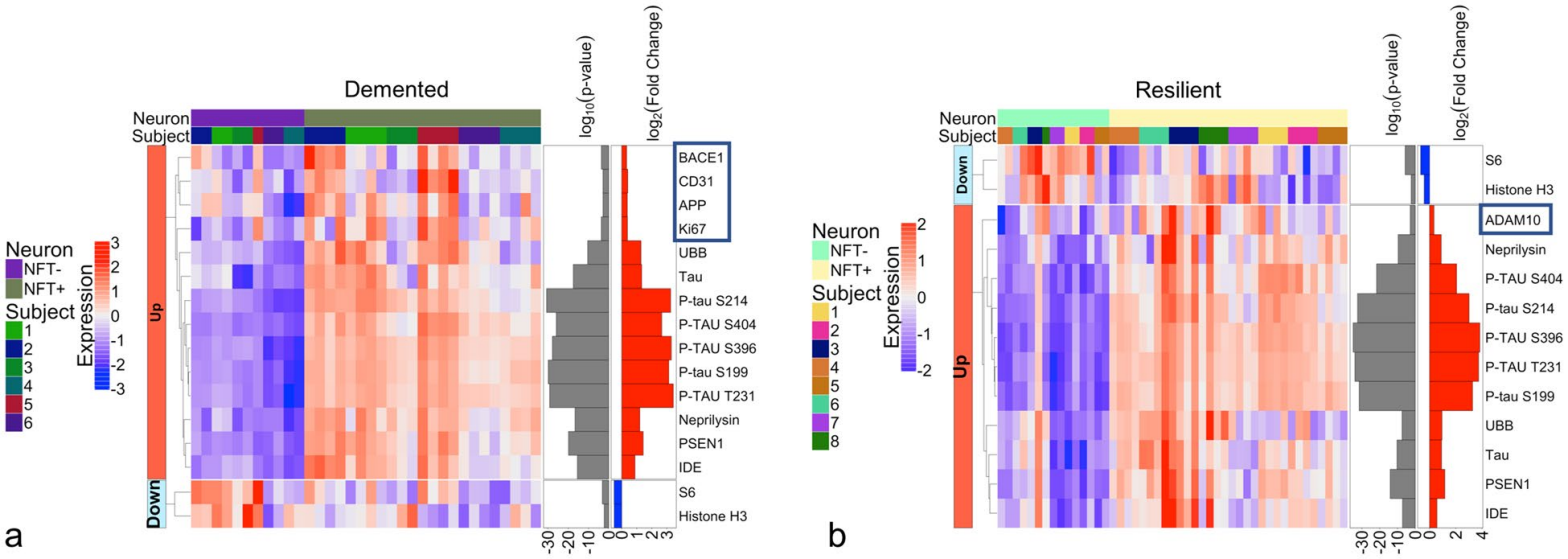
**a.** Heatmap of differentially expressed proteins when comparing NFT-bearing neurons to non-NFT-bearing neurons in demented only, **b.** NFT-bearing neurons to non-NFT-bearing neurons in resilient only (*p* < 0.01); FDR was used to adjust the p-values and it is represented as log10 (*p* value) in the figure with a threshold of 0.01

This same analysis was performed separately within each subgroup (demented and resilient) as well. The two subgroups have many overlapping differentially expressed proteins when comparing their NFT-bearing neurons to non-NFT-bearing neurons (Fig. 3a., Additional file 3a. and Fig. 3b., Additional file 4b.). However, the resilient do not have APP, BACE1 or Ki-67 upregulated in their NFT-bearing neurons as the demented do, yet they do have ADAM10 upregulated, whereas the demented do not have ADAM10 upregulated. This suggests that the resilient may develop tangles without subsequent changes in several deleterious proteins related to β-amyloid processing, but may have more of the protective proteins. In addition, Ki-67 is not increased in the resilient, which could suggest less entry into a senescence phenotype in the resilient.

**Fig. 4:**
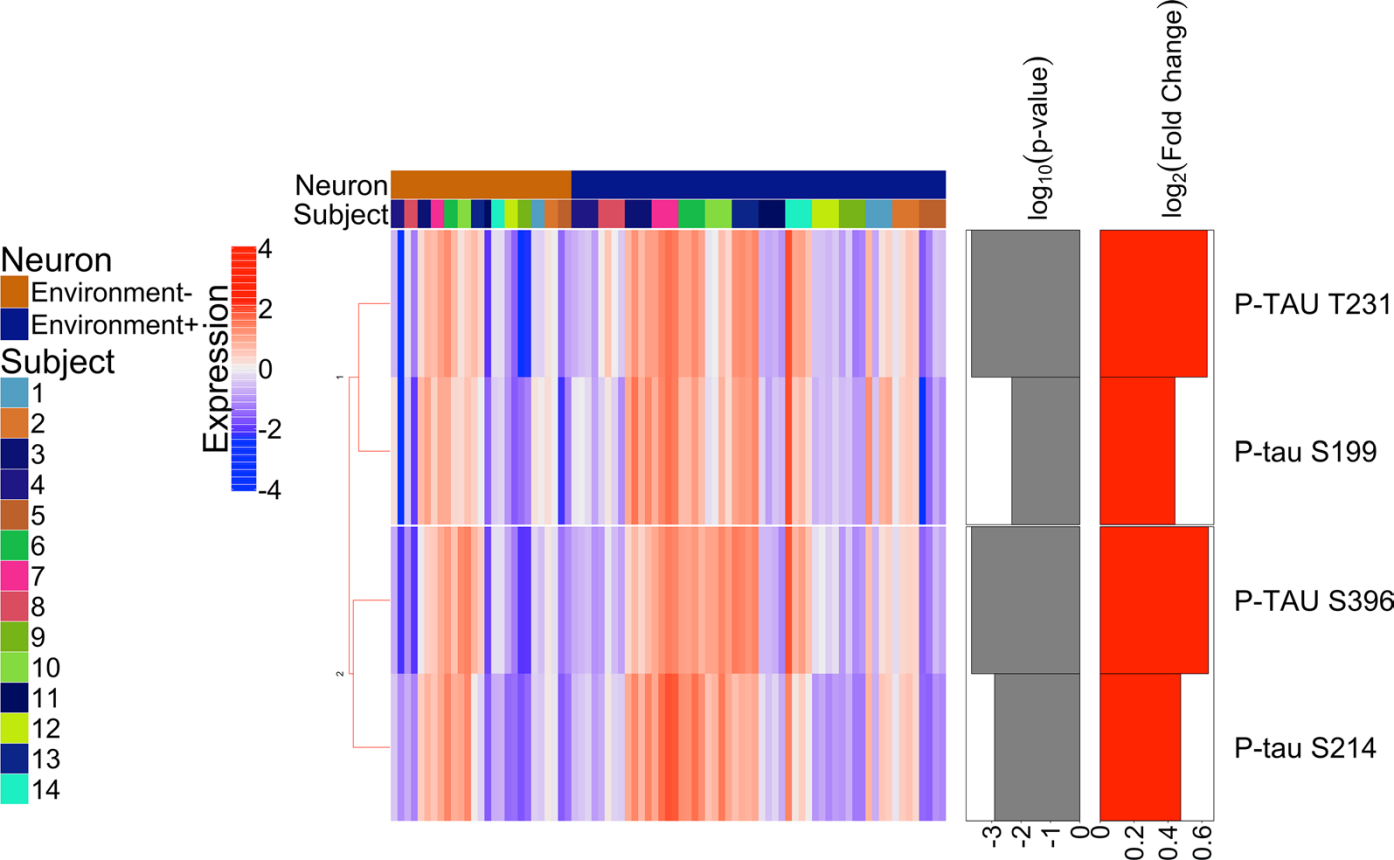
Heatmap of differentially expressed proteins when comparing the immediate neuronal microenvironments of NFTs to the microenvironment of normal non-tangle-bearing neurons (*p* < 0.01); FDR was used to adjust the p-values and it is represented as log10 (*p *value) in the figure with a threshold of 0.01

We also compared the immediate neuronal microenvironments of the NFT-bearing neurons and the non-NFT-bearing neurons, regardless of cognitive status. In this analysis we found that only levels of phosphorylated-tau differed (Fig. 4, Additional file 5); specifically, p-tau Thr231, Ser199, Ser396, and Ser214 were upregulated in the microenvironments of the NFTs. Of note, neither total tau nor Ser404 expression differed between the microenvironments indicating distinct spatial distributions among the elevated tau species.

## Resilient vs demented

**Fig. 5:**
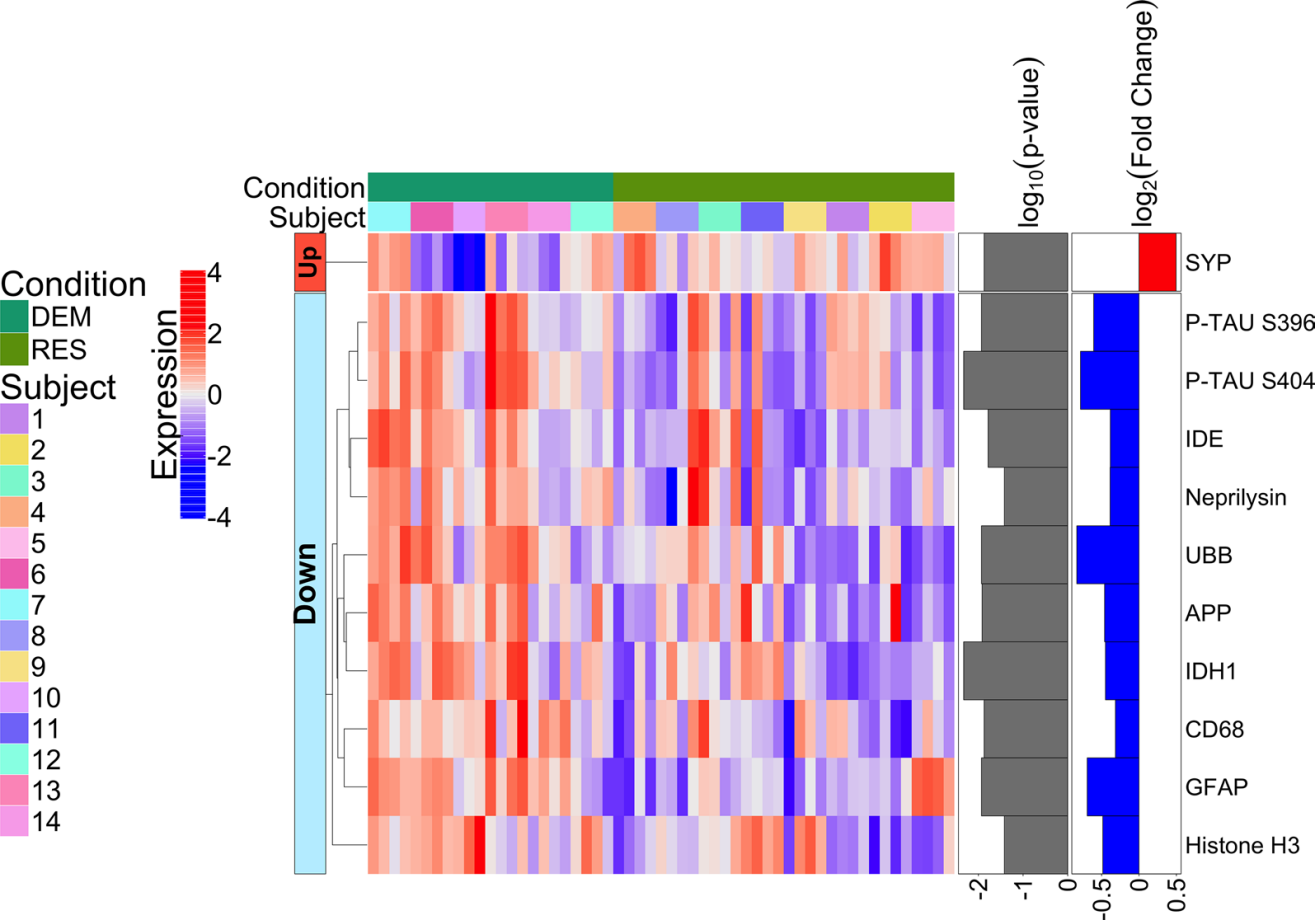
Heatmap of differentially expressed proteins when comparing NFT-bearing neurons of the resilient (RES, non-demented with AD pathology) to NFT-bearing neurons of the demented with AD pathology (DEM) (*p* < 0.05); FDR was used to adjust the p-values and it is represented as log10 (*p* value) in the figure with a threshold of 0.05

Our next set of analyses were designed to compare the resilient (AD pathology without dementia) to the demented (AD pathology with dementia). Comparing NFT-bearing neurons between resilient and demented, we identified 11 proteins with significantly different expression (Fig. 5, Additional file 6). Most of these displayed lower expression in the resilient individuals, and included proteins involved in β-amyloid processing such as APP, neprilysin, and IDE; proteins associated with proteostasis such as ubiquitin and p-tau; and proteins involved in neuroinflammation such as GFAP and CD68. Histone H3 expression was also found to be expressed at significantly lower levels in the resilient NFT-bearing neurons than NFT-bearing neurons from demented. In addition, tau phosphorylated at Serine 396 and 404 were lower in the tangles of the resilient. Interestingly, these two site-specific post-translational modifications of tau are thought to be more abundant in later stages of AD [39]. Furthermore, it has been reported that phosphorylation of tau at these 2 sites (S396 and S404) leads to inhibition of its normal function [17]. This raises the possibility that in avoiding phosphorylation at these sites, neurons in resilient individuals are able to maintain normal tau function and neuronal stability, despite the presence of tangles. Additional evidence supporting that NFT-bearing neurons from resilient cases may be better maintained was the observed increased expression of synaptophysin (SYP) in NFT-bearing neurons of the resilient individuals.

**Fig. 6:**
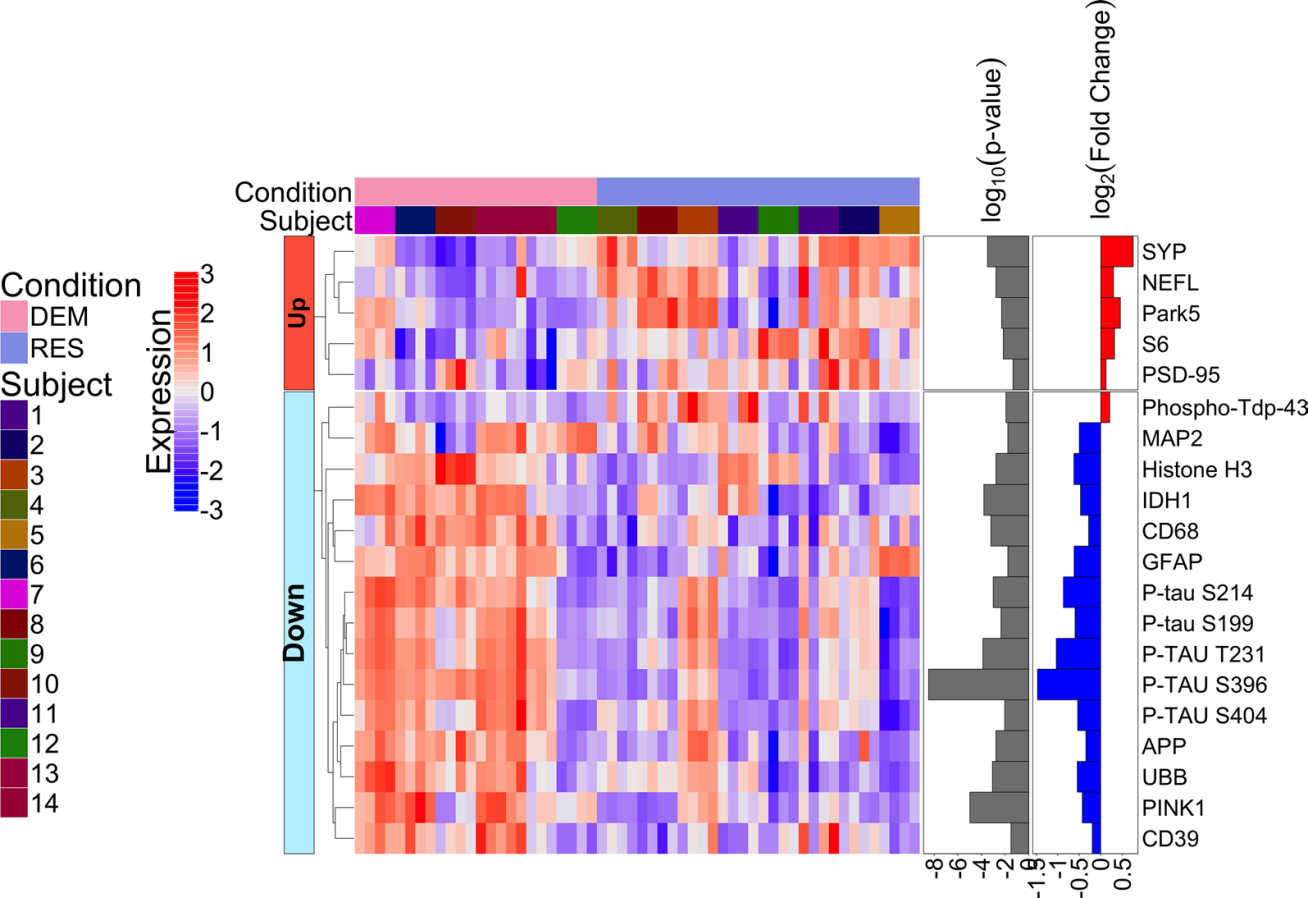
Heatmap of differentially expressed proteins when comparing the immediate neuronal microenvironment of NFT-bearing neurons in the resilient (RES) to the immediate neuronal microenvironment of NFT-bearing neurons in the demented with AD pathology (DEM) (*p* < 0.05); FDR was used to adjust the p-values and it is represented as log10 (p value) in the figure with a threshold of 0.05

We next identified proteins that displayed significantly different expression in the environment of the tangle-bearing neurons in the demented individuals as compared to the resilient (Fig. 6, Additional file 7). In this scenario, CD68, CD39 and GFAP were lower in the resilient (indicative of less inflammation). Other proteins with lower expression levels in the environment of NFT-bearing neurons of the resilient as compared to cases with dementia included PTEN induced kinase 1 (PINK1), a mitochondrial protein that is upregulated during cellular stress and unusually high energy demands [52], and NADP + -dependent isocitrate dehydrogenase 1 (IDH1), an enzyme critical to glucose metabolism and the primary producer of brain NADPH [3]. These proteins are generally upregulated in response to energetic or oxidative stress. Therefore, the resilient individuals likely maintain a lower burden of oxidative stress. The proteins with higher expression in the environment of the tangles in the resilient individuals as compared to the demented were proteins indicative of healthy neuronal structure and function (*i.e.,* synaptophysin, neurofilament light chain, and post-synaptic density protein 95 (PSD-95)). Park5, a ubiquitin c-terminal hydrolase important for stabilizing monomeric ubiquitin that has been reported to be decreased in AD brains [19], and ribosomal protein S6 were also higher in resilient cases compared to those with dementia. The increased abundance of these proteins suggests healthier intact axons, dendrites and synapses in the resilient.

**Fig. 7:**
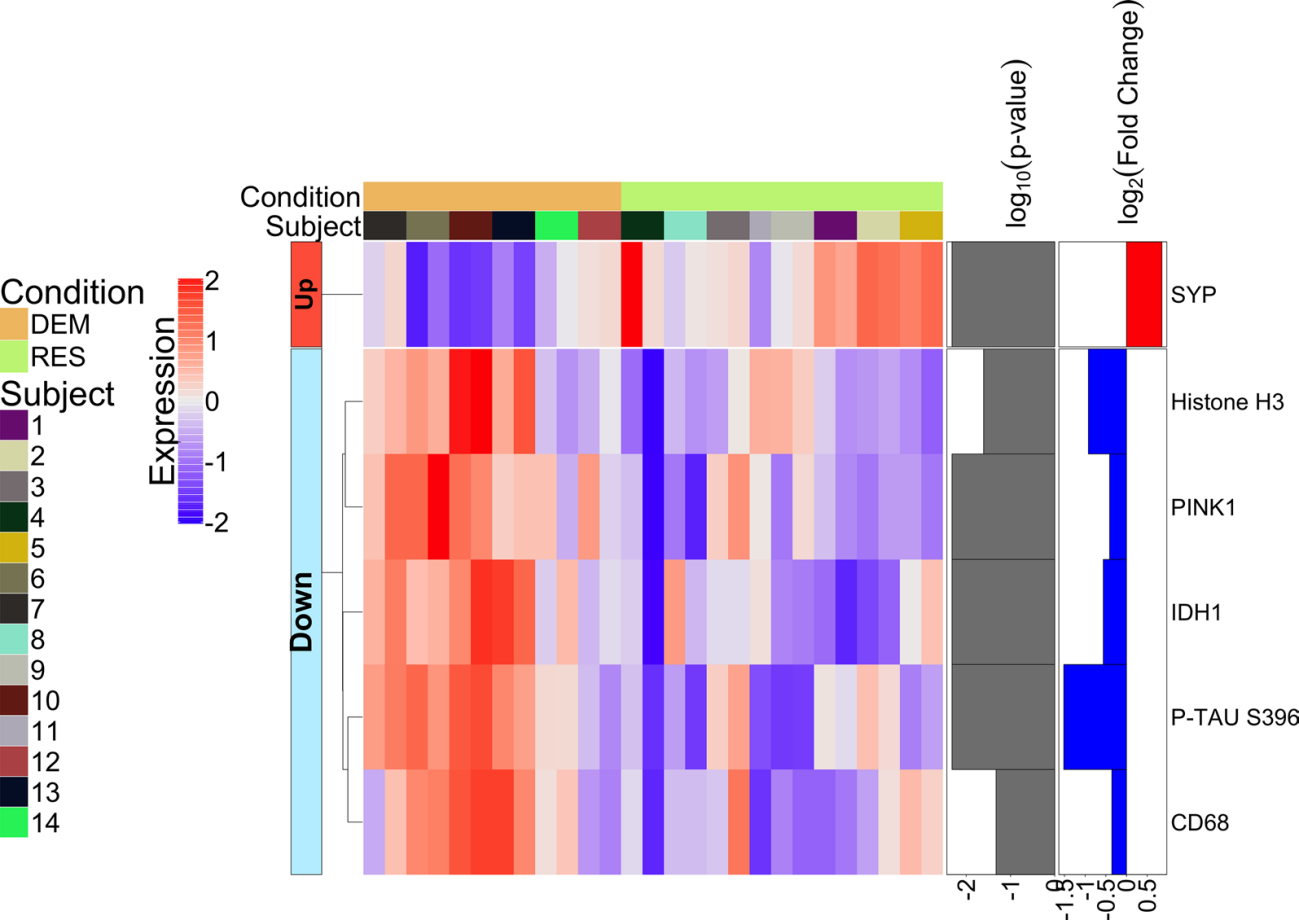
Heatmap of differentially expressed proteins when comparing the immediate neuronal microenvironment of non-NFT-bearing neurons in the resilient (RES) to the immediate neuronal microenvironment of non-NFT-bearing neurons in the demented with AD pathology (DEM) (*p* < 0.05); FDR was used to adjust the p-values and it is represented as log10 (*p* value) in the figure with a threshold of 0.05

When comparing the inner circle ROIs around non-tangle-bearing neurons of the demented and resilient (*i.e.,* the cell bodies of neurons without AT8-positive tau across cases), there were no significant differences in protein expression (Additional file 8a.). However, when comparing their immediate microenvironments, we observed significant differences whereby resilient cases displayed higher levels of synaptophysin, and lower levels of PINK1, IDH1, CD68, and p-tau S396 than demented cases (Fig. 7, Additional file 9b.). These data suggest that microenvironment differences may be impacting cognitive resilience, even in neurons without tau pathology.

## Additional observations

**Fig. 8:**
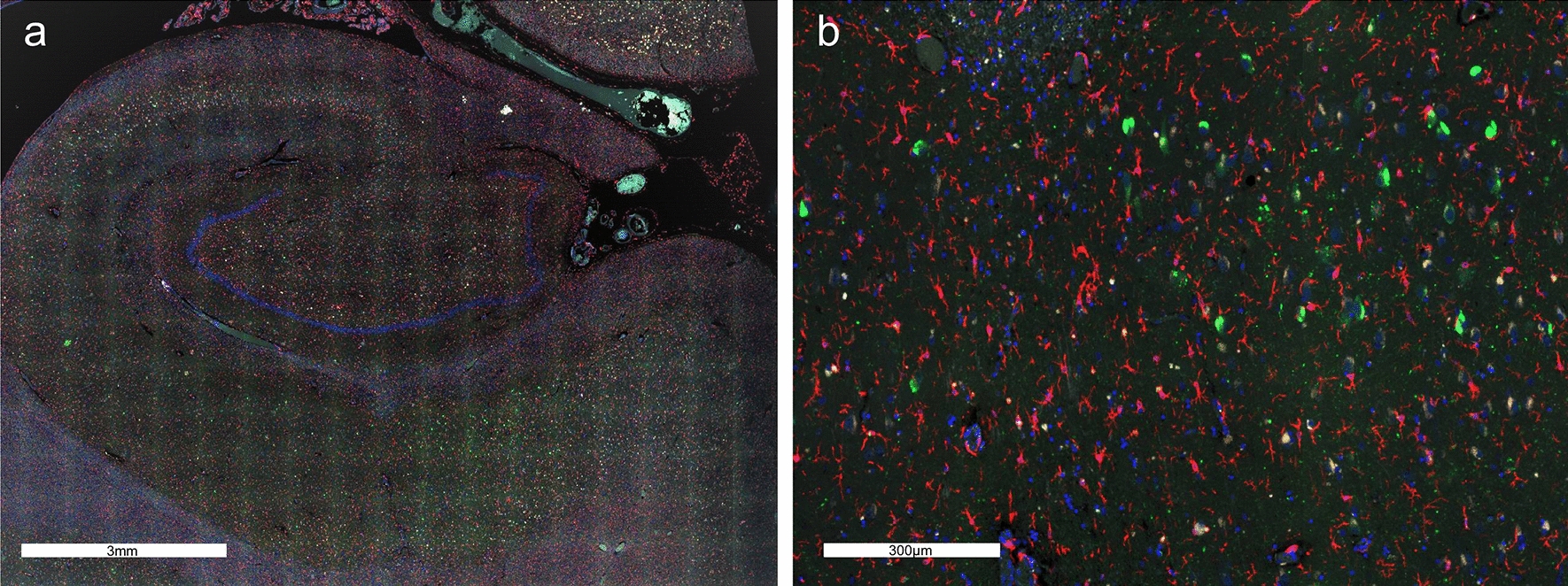
**a**. Hippocampus of a resilient case with abundant resting microglia, but a low abundance of plaques, scale bar represents 3 mm **b**. CA1 subregion of the same case with resting microglia and NFTs, but no plaques, scale bar represents 300 µm, sections were immunostained with the four morphology markers (AT8, green; β-amyloid, aqua; IBA-1, red; nuclear marker SYTO13, blue)

**Fig. 9:**
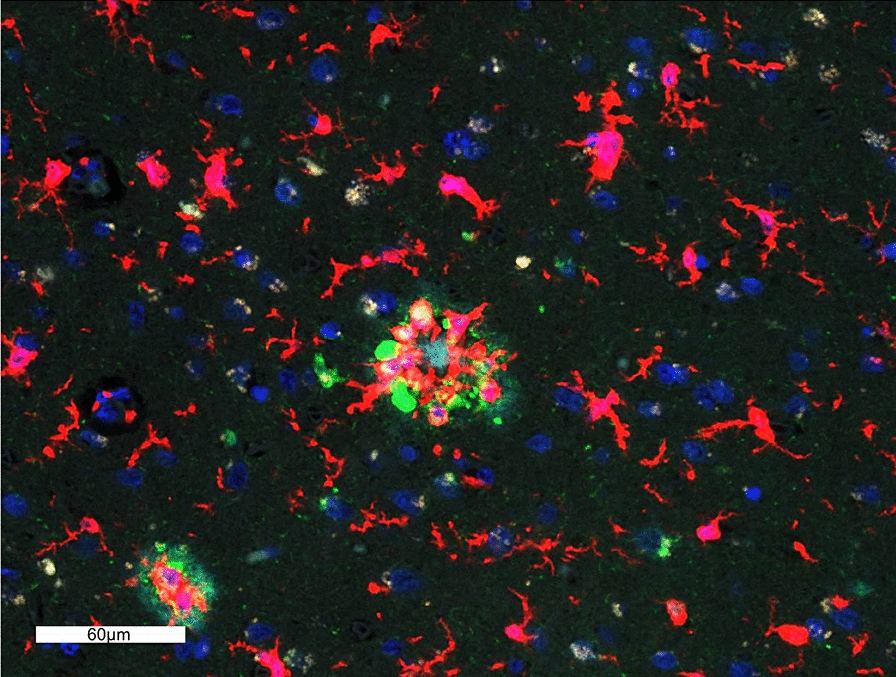
Neuritic plaque with activated microglia, scale bar represents 60 µm, sections were immunostained with the four morphology markers (AT8, green; β-amyloid, aqua; IBA-1, red; nuclear marker SYTO13, blue)

Upon review of the hippocampal sections stained with the morphology markers, we observed that the resilient harbored less neurofibrillary degeneration and plaque deposition overall in the hippocampal CA subregions as compared to the demented. In addition, we observed abundant quiescent (resting) microglia in the resilient hippocampi (Fig. 8), although there were frequent activated microglia attacking neuritic plaques in the fusiform cortex (Fig. 9).

Understanding that the average Braak stage is lower in the resilient subgroup (median Braak stage IV–V in the resilient vs. VI in the demented), we also analyzed protein expression differences in the NFTs of the all of the Braak stage IV cases vs. all of the Braak stage VI cases, regardless of cognitive status. The only significant differences in protein expression in the hippocampal NFTs of these 2 subgroups are p-tau S396 and S404 displaying higher expression in the Braak stage VI cases (two epitopes of p-tau that are observed in later stages of AD) [39, 48], which suggests that the protein differences associated with neuroinflammation and increased oxidative stress observed in the demented are not associated with the higher Braak stage in this instance, but more with cognitive status (Additional file 10).

## Discussion

Utilizing digital spatial profiling, we have demonstrated that neurofibrillary tangles not only express high levels of phosphorylated-tau as expected, but they also upregulate proteins related to β-amyloid processing. This raises the age-old “chicken or the egg” question of which comes first, the p-tau or the β-amyloid; our data suggest that it may be the NFT-bearing neurons that contribute more to Aβ production than neurons without phosphorylated-tau (*i.e.,* Fig. 2). NFT formation begins in the locus coeruleus in people in their teens to 20s (and all adults examined develop some degree of neurofibrillary degeneration in their lifetime) [9]. Whereas β-amyloid diffuse plaques begin in the neocortex in some people in their 40s-50s (but not all people develop plaques in their lifetime) [9]. The hippocampus is a nexus for the progression of both plaques and tangles. At Thal phase 2, plaques reach the entorhinal cortex and CA1 subregion of the hippocampus, then the molecular layer of the dentate gyrus at Thal phase 3, and the CA4 subregion at Thal phase 4. At Braak stage III, the CA1 subregion of the hippocampus harbors a substantial amount of neurofibrillary degeneration. Thus, the entorhinal cortex and hippocampus are where plaques and tangles initially “encounter” each other. It is difficult to say which comes first, however, our data may suggest that the NFTs in the demented are involved in instigating and/or regulating β-amyloid plaque deposition. Since it is known that plaques exist in the neocortex without tangles, this cannot be the case everywhere. Neuritic plaques, however, do appear only after neurofibrillary degeneration and plaque formation have coincided (first in the entorhinal cortex and hippocampus). Perhaps then, the demented have upregulated proteins within the NFTs that instigate the formation of neuritic plaques, rather than just diffuse plaques. Thal phase (stage of diffuse plaque deposition) does not correlate well with cognition, but CERAD neuritic plaque score *does* generally correlate well with cognition [53]. This raises the possibility that the demented are more inclined to form deleterious neuritic plaques, or vice versa.

It is true that only APP, neprilysin, and IDE display increased expression in the tangles of the demented when compared to the resilient, and neprilysin and IDE would both be protective. However, it is likely that the resilient do not need to upregulate neprilysin and IDE as much because they do not have APP upregulated in parallel. Also, when comparing the subgroups separately, the NFTs of the resilient did not upregulate BACE1 or APP. While these proteins are infamously known for their role in Aβ production and degradation; importantly, they also regulate other diverse cellular functions. One shared pathway among many of them is neuronal insulin sensitivity. Specifically, BACE1 expression levels inversely correlate with body weight, lipid and glucose homeostasis [31]; PSEN1 directly downregulates the expression of insulin receptor [28]; neprilysin upregulation is correlated with insulin resistance [56] while its decrease is associated with enhanced insulin sensitivity [61, 64]; IDE directly degrades insulin [34, 35] and is upregulated in brain cells with aberrant cell cycle activity [59]. These data build on prior reports showing aberrant insulin signaling in NFT-bearing neurons including phosphorylated insulin receptor substrate 1 (IRS1) at serine residues linked to insulin resistance [62]. Additionally, our data revealed a significant difference in synaptic proteins between neurons with or without NFT, which is a key feature of insulin resistant senescent neurons [11]. The activation of the cellular senescence stress response in NFT-bearing neurons was further supported in our dataset by aberrant Histone H3 and Ki-67 expression (Fig. 2).

In addition, we demonstrate that the areas of neurofibrillary degeneration in the resilient express lower levels of GFAP and CD68, indicative of less inflammation. These data suggest that lower levels of inflammation, despite neuropathology, may be contributing to cognitive resilience in the cases analyzed here. An increase in GFAP (or gliosis) generally occurs after neuronal loss. This aligns with observations of more neuronal loss in demented individuals, and suggests neuronal maintenance may be better achieved in the absence of GFAP and CD68 upregulation. Of note, we did not observe significant differences in microglia markers Iba1, P2ry12, TREM119, CD11b, CD163, CD40 and CD45, though we did observe lower CD68, commonly considered a marker of activated microglia, in the resilient. Together, these data suggest that total microglia may be comparable between resilient and demented, whereas activated microglia are more closely associated with dementia.

Furthermore, analyses revealed lower levels of proteins which respond to cellular stress or unusually high energy demands such as PINK1 and IDH1, in the resilient. PINK1 (PTEN-induced kinase) is a serine/threonine protein kinase that is upregulated in the mitochondria in times of cellular stress. IDH1 (isocitrate dehydrogenase) is an enzyme that catalyzes the oxidative decarboxylation of isocitrate to alpha-ketoglutarate, resulting in the production of NADPH from NADP + . IDH1 is upregulated under oxidative stress in order to limit oxidative damage [60]. This suggests there is a lower abundance of oxidative stress in the resilient. The resilient also displayed higher levels of synaptophysin, neurofilament light chain (NFL) and Park5. NFL has been reported to be increased in the CSF of AD patients, indicating degradation of neurons and their axons. However, if one has more NFL in the parenchyma, as the resilient do, it is indicative of healthier and intact axons. Park5 expression is lower in AD brains and an increased level of Park5 is thought to be protective for synaptic and cognitive function. Overall, there is less evidence of energetic and oxidative stress, lower neuroinflammation, and more evidence for healthy, intact axons and synapses in the resilient.

Although all but one of the resilient cases have sufficient pathology to qualify for a diagnosis of intermediate or high ADNC, they do display a lower abundance of neurofibrillary tangles and plaques in the hippocampus (by observation), and therefore harbor a lower total burden of AD pathology in the hippocampus. This could be significantly contributing to their superior cognition and cannot be overlooked. However, the differences we found in protein expression within the tangles (most notably molecular machinery critical to Aβ generation, insulin signaling, protein accumulation and cellular senescence) and their microenvironments demonstrate that there are multiple factors contributing to the ability of the resilient to protect themselves in the face of the neurofibrillary degeneration. These factors may also play a role in reducing the development of more widespread abundant AD pathology as would occur through increased Aβ production and cellular senescence-associated inflammation. It is also important to note that these cases do not have significant comorbid pathologies, as multiple studies have demonstrated that the presence of concomitant pathologies is common in AD, and these can significantly affect cognition [4, 24, 30].

Previous studies have examined transcriptomic gene expression changes within neurofibrillary tangles [13, 16, 29] and in AD brains [29], however many of the genes that are upregulated do not correlate well with protein expression changes. In the prefrontal cortex, GFAP gene expression was found to be upregulated in AD brains [29], and in CA1 pyramidal neurons gene expression of synaptophysin and PSD-95 were downregulated in individuals with MCI and AD [12], corroborating our findings of preservation of synaptophysin and PSD-95 protein expression in the resilient hippocampi. There have not been many published -omic studies investigating resilience. One genome-wide association study (GWAS) examined SNPs associated with resilience against amyloidosis and found differences in genetic drivers of bile acid homeostasis, vascular and metabolic risk factors, and neuropsychiatric conditions between resilient and demented individuals [15].

In addition, studies have used affinity purification mass-spectrometry (AP-MS) to investigate protein interactions with phosphorylated-tau [5, 14, 22, 32], identifying several proteins that overlap with our findings including TDP-43, MAP2, GFAP, UBB, and IDH. We found that UBB, GFAP, and IDH were lower in the NFTs of the resilient as compared to the demented, as were p-tau S396 and S404. Interestingly, Drummond et al. identified p-tau using PHF-1 which recognizes tau phosphorylated at S396 and S404 [14], and so their study may have preferentially found proteins that are upregulated at later stages of AD and primarily in the demented.

In light of the significant findings of this study, there are several limitations that should be noted. Firstly, we were limited in the number of cases (8) with sufficient clinical and pathologic data to be classified as “resilient” as truly resilient cases are quite rare in neurodegenerative and community-based brain banks and many post-mortem studies of dementia and resilience have fewer than 10 resilient cases [26, 43]. Additionally, more detailed correlations between MMSE scores and other factors were not possible to make with the present data, as a number of the “demented” cases did not have definitive MMSE values, only that they were < 19 to qualify as “demented” (Table 1). In addition, the number of regions of interest per slide was limited to 12. However, the DSP technology has a number of benefits over more traditional means. This methodology combines both spatial and quantitative capabilities, and as such can perform very granular quantification of proteins in well-defined, restricted areas, in an unbiased manner unlike traditional immunohistochemical staining, which can identify specific proteins, but does not add much quantitative value, and western blotting or proteomics mass spectrometry, which can provide accurate quantification but does not have the spatial localization ability of DSP.

## Conclusions

This spatial proteomic study of resilience in the face of AD neuropathologic change is a unique investigation providing novel findings that advance our understanding of the mechanisms by which some individuals are able to evade cognitive decline despite the presence of Alzheimer disease pathologic changes. By utilizing NanoString GeoMx™ DSP, we found that NFT-bearing neurons display increased expression of several proteins involved in β-amyloid processing, which reinforces the assumption that tangle and plaque pathology may develop synergistically in AD. However, there was significantly lower expression of β-amyloid processing proteins in the tangles of resilient individuals. In addition, the resilient individuals displayed lower expression of proteins involved in neuroinflammation, and a protein signature suggestive of an environment containing less energetic and oxidative stress, and more amenable to the maintenance of neuronal integrity and synaptic connections.

## Supplementary Information

**Additional file 1**: DSP antibody probes analyzed (86 proteins)

**Additional file 2**: Differential expression of proteins when comparing neurofibrillary tangle (NFT)-bearing neurons to normal non-NFT-bearing neurons

**Additional file 3**: Differential expression of proteins when comparing NFT-bearing neurons to non-NFT-bearing neurons in demented only

**Additional file 4**: NFT-bearing neurons to non-NFT-bearing neurons in resilient only

**Additional file 5**: Differential expression of proteins when comparing the immediate neuronal microenvironments of NFTs to the microenvironment of normal non-tangle-bearing neurons

**Additional file 6**: Differential expression of proteins when comparing NFT-bearing neurons of the resilient (non-demented with AD pathology) to NFT-bearing neurons of the demented with AD pathology

**Additional file 7**: Differential expression of proteins when comparing the immediate neuronal microenvironment of NFT-bearing neurons in the resilient (non-demented with AD pathology) to the immediate neuronal microenvironment of NFT-bearing neurons in the demented with AD pathology

**Additional file 8**: Differential expression of proteins when comparing normal (non-NFT-bearing neurons) of the resilient to non-NFT-bearing neurons of the demented with no significant differences

**Additional file 9**: Differential expression of proteins when comparing the immediate neuronal microenvironment of non-NFT-bearing neurons in the resilient to the immediate neuronal microenvironment of non-NFT-bearing neurons in the demented

**Additional file 10**: Differential expression of proteins when comparing NFT-bearing neurons of all Braak stage IV cases to NFTs of all Braak stage VI cases

## Abbreviations

AD: Alzheimer disease
ADAM10: A disintegrin and metalloproteinase domain-containing protein 10
ADNC: Alzheimer disease neuropathologic change
APP: Amyloid precursor protein
Aβ: Amyloid-β
BACE: Beta-secretase
CERAD: Consortium to Establish a Registry for Alzheimer Disease
CD68: Cluster of differentiation 68
CN: Cognitively normal
DEM: Demented
DSP: Digital spatial profiler
ERCC: External RNA Controls Consortium
FFPE: Formalin-fixed paraffin-embedded
GFAP: Glial fibrillary acidic protein
IBA-1: Ionized calcium binding adaptor molecule 1
IDE: Insulin degrading enzyme
IDH: Isocitrate dehydrogenase
IHC: Immunohistochemistry
NEFL (NFL): Neurofilament light chain
NFT: Neurofibrillary tangle
MMSE: Mini-metal state examination
Park5 (UCH-L1): Ubiquitin c-terminal hydrolase
PINK1: PTEN induced kinase
PSEN: Presenilin
RES: Resilient
ROI: Region of interest
SNP: Single nucleotide polymorphism
SYP: Synaptophysin
TDP: TAR DNA-binding protein
UBB: Ubiquitin

## Declarations

### Availability of data and material

All data generated or analyzed during this study are included in this published article and its supplementary information files.
